# Successful treatment of aberrant splenic artery aneurysm with a combination of coils embolization and covered stents

**DOI:** 10.1186/1471-2482-14-62

**Published:** 2014-08-30

**Authors:** Weimin Zhou, Jiehua Qiu, Qingwen Yuan, Wei Zhou, Jixin Xiong, Qingzhong Zeng

**Affiliations:** 1 Department of Vascular Surgery, The second affiliated hospital of Nanchang University, No 1#, Minde Road, Nanchang, China

**Keywords:** Aberrant splenic artery, Aneurysm, Covered stent, Coils

## Abstract

**Background:**

Aneurysms of an aberrant splenic artery originating from the superior mesenteric artery (SMA) are extremely rare; however, they are clinically important because possible rupture could be catastrophic. The methods of treatment for this condition include surgical resection, minimally invasive techniques (include laparoscopic technique) and endovascular therapy. The purpose of this study is to evaluate the efficacy of coils embolization combined with covered stents to treat aberrant splenic artery aneurysm (SAA).

**Cases Presentation:**

We report four consecutive cases of aberrant SAA that the aberrant splenic artery was embolized with coils and the superior mesenteric artery was excluded with a covered stent and an up-to-date review of all previous cases in the field. A follow-up computed tomography performed 6 to 12 months postoperatively showed persistent exclusion with marked shrinkage of the aneurysm sac.

**Conclusions:**

The authors believe although early results are promising, further careful follow-up will be needed to determine the long-term clinical efficacy, safety and applicability of this approach.

## Background

Aneurysms of an aberrant splenic artery originating from the superior mesenteric artery (SMA) are extremely rare; however, they are clinically important because possible rupture could be catastrophic. Since Ghatan et al. [1] reported the first case in 1967, we have only identified 31 cases reported in the English medical literature [1-21] via a Medline database search from 1966 to August 2013 with key words “aneurysms and aberrant splenic artery or anomalous splenic artery”. The methods of treatment for this condition include surgical resection, minimally invasive techniques (include laparoscopic technique) and endovascular therapy. We present here four consecutive cases of aberrant splenic artery aneurysm (SAA) that were successfully treated with a combination of coils embolization and covered stent implantation between May 2012 to December 2013. This appears to be a promising minimally invasive approach in managing this rare entity. The purpose of this study is to review the literature and report our experience.

## Cases report

**Figure 1:**
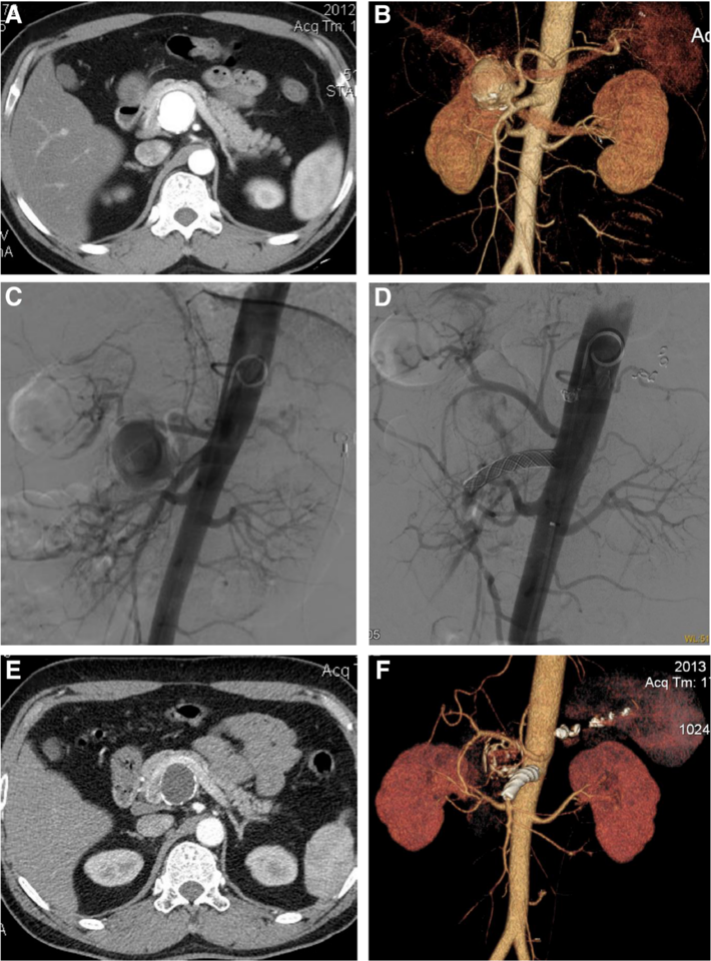
**The image finding of case one during preoperation, intraoperation and follow-up. A** and **B**, Preoperative computed tomographic angiography (CTA) confirmed a 40 mm × 38 mm splenic artery aneurysm (SAA) near the origin of the superior mesenteric artery (SMA). **C**, Intraoperative digital subtraction angiogram (DSA) demonstrated the findings same as CTA. **D**, The completion aortogram revealed disappearance of the aberrant SAA lumen and patency of the SMA but a tiny endoleak. **E** and **F**, Follow-up abdominal CT scan in 12 months postoperatively confirmed an occluded aneurysm sac with marked shrinkage and patency of the SMA.

**Figure 2:**
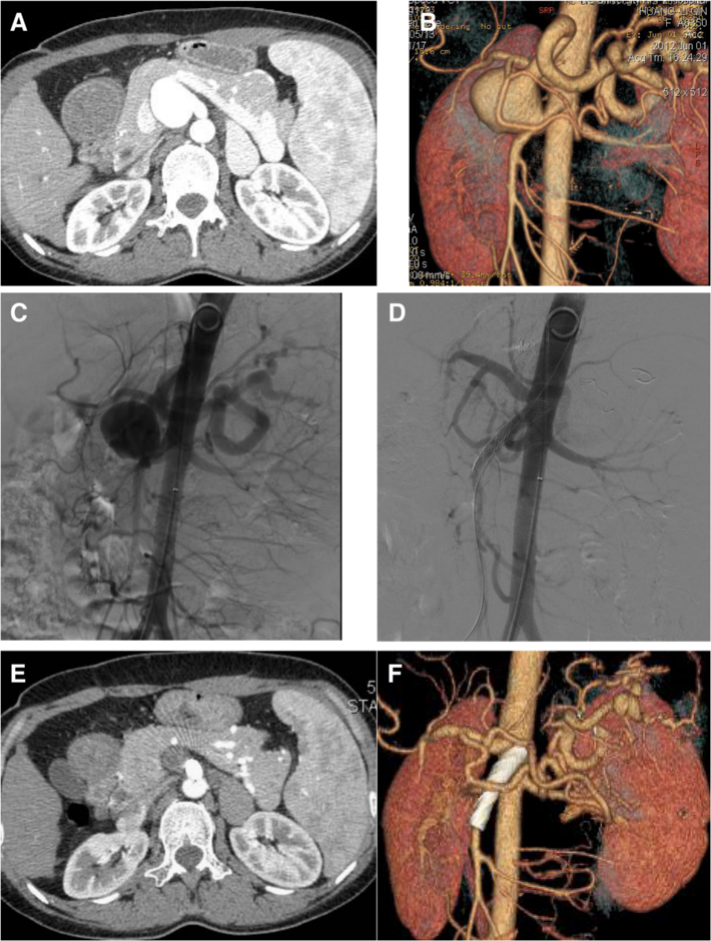
**The image finding of case two during preoperation, intraoperation and follow-up. A, B** and **C**, Preoperative CTA and intraoperative DSA demonstrated a 36 mm × 32 mm SAA near the origin of the SMA and the proximal caliber of the SAA was 11 mm. **D**, The completion aortogram revealed no evidence of endoleak; disappearance of the aberrant SAA lumen and patency of the SMA and the branches immediately after the stent deployment. **E** and **F**, Follow-up abdominal CT scan in 12 months postoperatively confirmed an occluded aneurysm sac with marked shrinkage and patency of the SMA.

**Figure 3:**
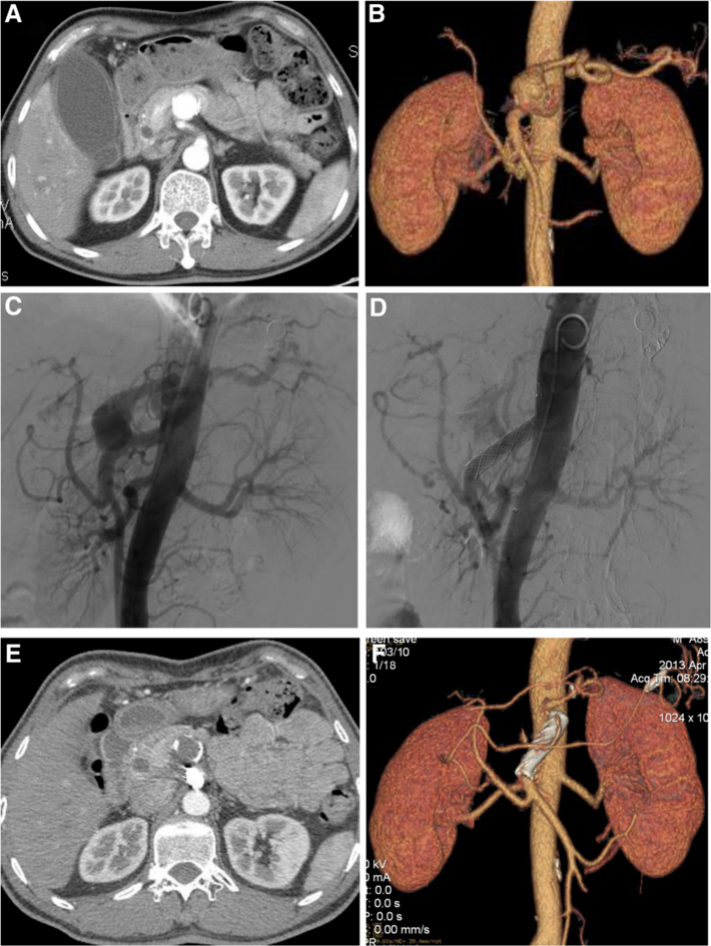
**The image finding of case three during preoperation, intraoperation and follow-up. A**, **B** and **C**, Preoperative CTA and DSA demonstrated a 35 mm × 34 mm SAA near the origin of the SMA and the proximal neck of the SAA was 5 mm. **D**, The completion aortogram revealed evidence of a small endoleak; disappearance of the aberrant splenic artery aneurysms lumen and patency of the SMA. **E** and **F**, The CTA in 10 months postoperatively demonstrated that the SAA sac was thrombosed with marked shrinkage; the SMA is patent with no infarction or abscess formation in the spleen.

**Figure 4:**
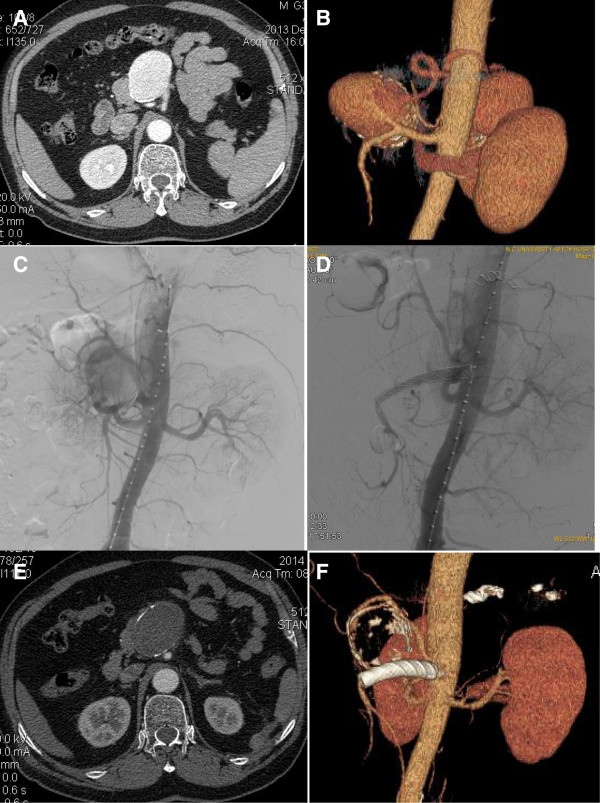
**The image finding of case four during preoperation, intraoperation and follow-up. A**, **B** and **C**, Preoperative CTA and DSA demonstrated a 49 mm × 59 mm SAA near the origin of the SMA and the proximal neck of the SAA was 5 mm. **D**, The completion aortogram revealed no evidence of endoleak; disappearance of the aberrant splenic artery aneurysms lumen and patency of the SMA. **E** and **F**, The CTA in 6 months postoperatively demonstrated that the SAA sac was thrombosed with marked shrinkage; the SMA is patent with no infarction or abscess formation in the spleen.

Case 1. A 37-year-old male patient was incidentally found to have a visceral aneurysm on abdominal ultrasound when he had a routine physical examination. A computed tomographic angiography (CTA) confirmed a 40 mm × 38 mm SAA near the origin of the SMA (Figure 1A,B). The aneurysm arose off the superior-lateral margin of the SMA and projected anteriorly and to the right. The SMA was widely patent and angiographic canalization of the celiac trunk did not reveal any branches supplying the spleen. The treatment procedure was performed under local anesthesia. A 6-French catheter sheath was cannulated from the right femoral artery and a pigtail angiographic catheter was put into the abdominal aortic artery via a 0.035 inch guidewire. A digital subtraction angiogram (DSA) showed an aneurysm at the root of an aberrant splenic artery that was arising 3 cm distal to the origin of the SMA and the distal splenic artery (SA) diameter being 6 mm (Figure 1C). Percutaneous transabdominal catheterization of the aneurysm was performed to cannulate the distal splenic artery as well as deposit coils. The distal splenic artery was cannulated through the lumen of the aneurysm. The catheter was then pulled back to the mid-splenic artery. Two 6 mm and one 8 mm coils were placed in the splenic artery. Two additional 6 mm coils were placed into the 8 mm coils to effect occlusion of the mid splenic artery. A repeated injection of contrast dye showed occlusion of the mid splenic artery. The left brachial artery was then punctured and cannulated with a 6-French catheter sheath from which a 5 F vertebral catheter was inserted into the abdominal aortic artery via a 0.035 inch guidewire because of the small angle of SMA. On sub-selective catheterization of the SMA we have also used 5 F vertebral catheter and curved Terumo guidewire (stiff type, Terumo Corporation, Japan). The covered stent (8 mm × 40 mm, Wallgraft, Boston Scientific, USA)was cannulated with a 6-French delivery introducer sheath via the 0.035 inch Terumo stiff type guidewire through the left brachial artery (Figure 1D). The patient remained asymptomatic, apyrexial and haemodynamically stable after the procedure and was discharged after 48 h of observation. A CT scan of the abdomen 7 days later demonstrated the aberrant splenic artery aneurysm sac was full of thrombi with a tiny endoleak. Follow-up abdominal CT scan in 12 months postoperatively confirmed an occluded aneurysm sac with marked shrinkage and patency of the SMA (Figure 1E,F). There was not any infarction or abscess formation in the spleen. No fever and the inflammatory markers were normal.Case 2. A 36-year-old female patient was admitted to our center just when the first case was discharged. She was diagnosed with mesenteric artery aneurysm at the outpatient department. But the CTA demonstrated a 36 mm × 32 mm splenic artery aneurysm near the origin of the SMA and the proximal caliber of the SAA was 11 mm (Figure 2A,B). The root of the aberrant splenic artery aneurysm was arising at 2 cm distal to the origin of the SMA. The treatment procedure was same as in Case one. Three 8 mm and one 10 mm coils were placed in the splenic artery. The covered stent (10 mm × 40 mm, Wallgraft, Boston Scientific, USA)was cannulated with a 10-French delivery introducer sheath via the 0.035 inch Terumo stiff type guidewire through the left brachial artery. The completion aortogram revealed no evidence of endoleak; disappearance of the aberrant splenic artery aneurysms lumen and patency of the SMA and the branches immediately after the stent deployment (Figure 2C,D). The patient was discharged in 48 h postoperatively and CTA demonstrated the aneurysm sac was full of thrombi with no endoleak on postoperative Day 9. Follow-up abdominal CT scan in 12 months postoperatively confirmed an occluded aneurysm sac with marked shrinkage (Figure 2E,F). There was no infarction or abscess formation in the spleen. No fever and the inflammatory markers were normal.Case 3. A 52-year-old male patient was found to have a visceral aneurysm on abdominal CT when he was diagnosed with gallbladder stone at a routing physical examination. CTA also demonstrated a 35 mm × 34 mm splenic artery aneurysm near the origin of the SMA and the proximal neck of the SAA was 5 mm (Figure 3A,B). The root of the aberrant splenic artery aneurysm was arising 30 mm distal to the origin of the SMA. The treatment procedure was same as in Case one and Case two. Three 7 mm and two 8 mm coils were placed in the splenic artery. The covered stent (10 mm × 50 mm, Wallgraft, Boston Scientific, USA)was cannulated with a 10-French delivery introducer sheath via the 0.035 inch Terumo stiff type guidewire through the left brachial artery. The completion aortogram revealed evidence of a small endoleak; disappearance of the aberrant splenic artery aneurysms lumen and patency of the SMA and the branches immediately after the stent deployment (Figure 3C,D). He was transferred to the Department of Hepatobiliary Surgery to perform laparoscopic cholecystectomy three days postoperatively. No complaint or complication was documented during 10 months follow-up. The CTA demonstrated that the splenic artery aneurysm sac was thrombosed with marked shrinkage; the SMA is patent with no infarction or abscess formation in the spleen (Figure 3E,F). No fever and the inflammatory markers were normal.Case 4. A 73-year-old male patient was found to have a splenic artery aneurysm on abdominal CTA. The CTA demonstrated a 49 mm × 59 mm splenic artery aneurysm near the origin of the SMA and the proximal neck of the SAA was 5 mm (Figure 4A,B). The root of the aberrant splenic artery aneurysm was arising 30 mm distal to the origin of the SMA. The treatment procedure was same as above 3 cases. Two 5 mm and two 8 mm coils were placed in the splenic artery. The covered stent (10 mm × 50 mm, Wallgraft, Boston Scientific, USA)was cannulated with a 10-French delivery introducer sheath via the 0.035 inch Terumo stiff type guidewire through the left brachial artery. The completion aortogram revealed no evidence of endoleak; disappearance of the aberrant splenic artery aneurysms lumen and patency of the SMA and the branches immediately after the stent deployment (Figure 4C,D). The patient was discharged and CTA demonstrated the aneurysm sac was full of thrombi with no endoleak on postoperative Day 5. No complaint or complication was documented during 6 months follow-up. The CTA demonstrated that the splenic artery aneurysm sac was thrombosed with marked shrinkage; the SMA is patent with no infarction or abscess formation in the spleen (Figure 4E,F). No fever and the inflammatory markers were normal.

### Review of the literature

Thirty-one cases of aberrant splenic artery aneurysms have been reported (Table 1). Of these, 11 cases were asymptomatic, 8 cases presented with epigastric pain, 2 cases presented with back pain, 2 cases presented with mild pain at the right hypochondrium, and 1 case presented with haematemesis and melaena. Their ages ranged from 29 to 73 years, with a median of 49 years. 10 patients were male, 21 patients were female. The median size of the aneurysm was 3.1 cm (range, 2-6 cm). Of the 31 cases reported, 18 (58%) were treated surgically and 12 (39%) were treat with endovascular. 1 case refused to treatment. Of the surgical treated patients, 16 cases were performed with aneurysmectomy (2 cases combined with splenectomy and 5 cases reconstruction of the SMA). Of the 12 cases of endovascular treated patients, 9 cases underwent coils embolization, 3 cases were performed stent graft placement and 1 case underwent coils embolization combined with stent graft placement. The duration time of follow-up is between 6 days to 1.8 years (mean 4.6 months).

**Table 1:** Summary of treatment for aberrant splenic artery aneurysm

***Authors***, ***Year***	***n***	***Size of aneurysm (cm)***	***Age (y)***	***Gender***	***Symptoms***	***Treatment***	***Follow***-***up***
Ghatan [1], 1967	1	4	58	Female	Left lumbar and flank pain	Aneurysmectomy	Unknown
Sidhu [2], 1995	1	2	35	Female	Asymptomatic	Aneurysmectomy	Unknown
Settembrini [3], 1996	2	4	45	Male	Mild pain at the right hypochondrium;	Aneurysmectomy and splenectomy;	12 months
		3.5	43	Female	Epigastric pain	Aneurysmectomy	12 months
Patel [4], 1998	1	2	59	Female	Unexplained episode of sepsis and rigors	Aneurysmectomy and reconstruction of the SMA	6 months
Pillay [5], 2003	1	Unknown	51	Male	Haematemesis and melaena	Coils embolization	6 months
Feo [6], 2004	1	4.3	64	Male	back pain	Aneurysmectomy with direct SA to SMA anastomosis (end to side)	3 months
Mastracci [7], 2005	2	2.7	31	Female	Intermittent severe epigastric pain radiating into her back;	Coils embolization and laparoscopic occlusion of the splenic artery;	9 months
		3.3	42	Female	Epigastric pain	Same as above	8 months
Migliara [8], 2005	2	2.5	50	Female	Asymptomatic;	Aneurysmectomy;	10 days
		2.5	47	Male	Asymptomatic	Coils embolization	6 days
Tochii [9], 2005	1	2.7	64	Female	Asymptomatic	Aneurysmectomy	17 days
Sato [10], 2006	1	2.5	50	Female	Discomfort at the epigastria	Detachable coils	1.8 yaers
LaBella [11], 2006	1	3	29	Female	Persistent bilateral lower quadrant pain	Ligation	Unknown
Facy [12], 2006	1	3	36	Male	Asymptomatic	Ligation	Unknown
Illuminati [13], 2007	1	2	51	Female	Nonspecific abdominal discomfort;	Aneurysmectomy	6 months
Liu [14], 2009	6	3.3	71	Female	Regurgitation and eructation;	Endovascular: stent graft placement	6 months
		3	52	Female	Asymptomatic;	Refuse to treatment	lost follow-up
		6	48	Female	Epigastric pain;	Endovascular: coils embolization with gelfoam and glue;	9 months
		3	52	Female	Asymptomatic;	Aneurysmectomy with splenectomy;	1 months
		3	38	Male	Asymptomatic;	Aneurysmectomy and patency of the splenic artery via collaterals;	6 months
		4.8	37	Male	Asymptomatic	Endovascular: stent graft	1 months
						placement	
Tanigawa [15], 2009	1	3.4	45	Male	Asymptomatic	Coil embolization	3 months
De Cloedt [16], 2010	1	2.3	41	Female	Epigastric pain	Aneurysmectomy	6 weeks
Jiang [17], 2011	1	2.1	67	Female	Intermittent epigastric pain	Endovascular stent-graft placement and coil embolization	12 months
Taneja [18], 2011	1	2.4	34	Male	Nonspecific upper abdominal pain	Balloon-mounted covered stent	6 months
Shu [19], 2011	3	3.9	73	Male	Asymptomatic;	Aneurysmectomy and reconstruction of the SMA; Aneurysmectomy and reconstruction of the SMA; Aneurysmectomy and reconstruction of the SMA	2 months
		4.3	54	Female	Abdominal pain;		10 days
		3	60	Female	Mild pain at the right hypochondrium		36 months
Borioni [20], 2013	1	2	53	Female	Epigastric pain	Implantation of multiple coils and an Amplatzer Vascular Plug	2 months
Wong [21], 2013	1	2.6	40	Female	Asymptomatic	Surgical resection with preservation of the spleen	Unknown

## Discussion

Visceral artery aneurysms are rare but important vascular disease because of their potential for fatal rupture. In general, treatment of visceral artery aneurysms is considered for patients with the size >2.0 cm in diameter, or rapid growth in aneurysm size, or with symptoms attributable to the aneurysm and for women of childbearing age [22]. SAAs are the most common visceral artery aneurysms (58% [23] to 70% [22]). An aberrant splenic artery arising from the SMA, also known as the splenomesenteric trunk, is a rare anatomical variant seen in less than 1% of the population [2,24]. In the process of embryogenesis, mislocation interrupt, incomplete interrupt or without interrupt of ventral longitudinal anastomosis may be the embryological mechanism of aberrant splenic artery. Aneurysms arising from an aberrant splenic artery are seen in a proximal position close to its origin [14]. This contrasts with the usual described location of these aneurysms in the mid to distal splenic artery in patients with normal anatomical origin of the artery [25,26]. The proximal anatomical location makes the management of these aneuryms more challenging. Methods of treatment of this condition include surgical resection, minimally invasive techniques (include laparoscopic techniques) and endovascular therapy. Compare with open surgery, laparoscopic techniques and endovascular therapy, total endovascular treatment is safe and minimally invasive with rapid recovery. In our cases, coils embolization alone was excluded as coils migration into the SMA may occur and result in intestinal ischemia due to the high blood flow within the aneurysms, wide proximal caliber and short proximal neck of the SAA. The tortuous SA was angled to SMA and the short proximal neck of the SAA made it difficult to place a cover stent to SMA and SA. Given backflow of blood from other supplying arteries of the spleen, we did not select SMA cover stent placement alone. Jiang and colleagues [17] reported a successful endovascular stent-graft placement and coil embolization in a 67-year-old woman suffered from an anomalous SAA. We thought that this method might be the optimal treatment option for this rare entity. After 6 to 12 months follow-up, the splenic artery aneurysm sac was thrombosed with marked shrinkage and the SMA remained patent; no endoleak, any infarction of the spleen or abscess formation because the SA has sufficient backflow from the short gastric artery. Our successful treatment in 4 consecutive patients suggests that the combined coils embolization and cover stent implantation may be a valuable therapeutic alternative when treating this rare lesion. To our knowledge, this is the first report of a combined coils embolization and covered stents implantation for the treatment of 4 or more consecutive cases of this condition.

## Conclusions

The authors believe that a combined coils embolization and covered stent implantation is a promising approach that seems to provide an effective, safe and minimally invasive option for the treatment of this very rare condition. Although early results are promising, further careful follow-up will be needed to determine the long-term clinical efficacy, safety and applicability of this technique.

### Consent

Written informed consent was obtained from the patients for publication of these cases report and any accompanying images. A copy of the written consent is available for review by the Editor-in-Chief of this journal.

## Competing interests

The authors declare that they have no competing interests.

## Authors’ contributions

WZ analyzed and interpreted the patient data. JQ, QY, Wei Zhou, JX and QZ were the major participants of the operation. All authors read and approved the final manuscript.

## Pre-publication history

The pre-publication history for this paper can be accessed here:

http://www.biomedcentral.com/1471-2482/14/62/prepub
